# Uncovering phenotypes of poor-pitch singing: the Sung Performance Battery (SPB)

**DOI:** 10.3389/fpsyg.2013.00714

**Published:** 2013-10-18

**Authors:** Magdalena Berkowska, Simone Dalla Bella

**Affiliations:** ^1^Department of Cognitive Psychology, University of Finance and Management (WSFiZ) in WarsawWarsaw, Poland; ^2^Movement to Health Laboratory (EuroMov), University of Montpellier 1Montpellier, France; ^3^Institut Universitaire de FranceParis, France

**Keywords:** music cognition, music disorders, singing, tone-deafness, amusia

## Abstract

Singing is as natural as speaking for humans. Increasing evidence shows that the layman can carry a tune (e.g., when asked to sing a well-known song or to imitate single pitches, intervals and short melodies). Yet, important individual differences exist in the general population with regard to singing proficiency. Some individuals are particularly inaccurate or imprecise in producing or imitating pitch information (poor-pitch singers), thus showing a variety of singing phenotypes. Unfortunately, so far there is not a standard set of tasks for assessing singing proficiency in the general population, allowing to uncover and characterize individual profiles of poor-pitch singing. Different tasks and analysis methods are typically used in various experiments, making the comparison of the results across studies arduous. To fill this gap we propose here a new tool for assessing singing proficiency (the Sung Performance Battery, SPB). The SPB starts from the assessment of participants' vocal range followed by five tasks: (1) single-pitch matching, (2) pitch-interval matching, (3) novel-melody matching, (4) singing from memory of familiar melodies (with lyrics and on a syllable), and (5) singing of familiar melodies (with lyrics and on a syllable) at a slow tempo indicated by a metronome. Data analysis via acoustical methods provides objective measures of pitch accuracy and precision in terms of absolute and relative pitch. The SPB has been tested in a group of 50 occasional singers. The results indicate that the battery is useful for characterizing proficient singing and for detecting cases of inaccurate and/or imprecise singing.

## Introduction

The majority in the general population can easily carry a tune. This ability, widespread and universal (Mithen, 2006), is as natural as speaking (Dalla Bella et al., 2007; Pfordresher and Brown, 2007; Dalla Bella and Berkowska, 2009). Occasional singing does not require particular musical tutoring or vocal training. Adult singing is typically accurate in terms of pitch and time (Dalla Bella et al., 2007; Dalla Bella and Berkowska, 2009), but not necessarily precise (Pfordresher et al., 2010). This observation contrasts with the general tendency to underestimate the abilities of the layman to sing in tune and in time. For example, when asked to estimate their singing proficiency, about 60% of a sample of more than 1000 university students judged that they cannot accurately imitate melodies (Pfordresher and Brown, 2007). Yet, when singing proficiency is systematically assessed in the lab, it turns out that around 85–90% of occasional singers can sing in tune (Dalla Bella et al., 2007; Pfordresher and Brown, 2007; Dalla Bella and Berkowska, 2009; lower estimates, however, are reported using a stricter criterion, Hutchins and Peretz, 2012, or when considering precision instead of accuracy, Pfordresher et al., 2010).

This common ability is underpinned by a complex neuronal network (i.e., the song system), which involves perceptual processes, motor planning and control, auditory-motor matching and memory processes (e.g., the “vocal sensorimotor loop,” VSL; see Berkowska and Dalla Bella, 2009b; Dalla Bella et al., 2011, for reviews). The song system can break down as a result of brain damage or neurogenetic (i.e., congenital) disorders, thereby leading to poor-pitch singing (e.g., Ackermann et al., 2006; Pfordresher and Brown, 2007; Dalla Bella et al., 2009; Stewart et al., 2009; Hutchins and Peretz, 2012). In this article we will focus on singing in healthy individuals in absence of brain damage. Approximately 10–15% of the population without musical training is inaccurate when singing well-known melodies from memory or when imitating pitch sequences (Dalla Bella et al., 2007; Pfordresher and Brown, 2007; Dalla Bella and Berkowska, 2009). Poor-pitch singing, often considered as a sign of a general lack of musicality (Sloboda et al., 2005), can be associated to impoverished pitch perception, as in the case of “congenital amusia” (Ayotte et al., 2002; Peretz and Hyde, 2003). Together with poor-pitch singing (Dalla Bella et al., 2009), individuals with congenital amusia have difficulties in recognizing familiar tunes and in pitch discrimination (Ayotte et al., 2002; Hyde and Peretz, 2004). Interestingly, poor pitch perception is not always accompanying inaccurate singing. Poor-pitch singing can co-exist with unimpaired pitch perception (Bradshaw and McHenry, 2005; Dalla Bella et al., 2007; Pfordresher and Brown, 2007; Wise and Sloboda, 2008), as in “purely vocal tone deafness” (Dalla Bella et al., 2007). The opposite dissociation (i.e., spared vocal performance in the presence of deficient perception), although more counterintuitive, was also described. For example, congenital amusics who are unable to detect pitch direction (i.e., judge whether the second tone of a pair is higher or lower than the first) can still imitate the correct pitch direction (Loui et al., 2008; Dalla Bella et al., 2009).

The distinctions among poor-pitch singers are not limited to the reported dissociation between perception and action. For example, in a preliminary study (Dalla Bella and Berkowska, 2009) the renditions of 39 occasional singers when they produced from memory and imitated well-known songs (e.g., Brother John, Jingle Bells) were measured in terms of accuracy for absolute pitch (i.e., the amount of pitch transposition) and relative pitch (i.e., accuracy in reproducing pitch intervals). Participants were qualified as poor-pitch singers in term of absolute or relative pitch when their performance departed from at least 2 *SD* from the group average. Poor-pitch singing in some cases selectively affected absolute pitch (8% of the occasional singers transposed pitch by more than 4 semitones without revealing inaccurate interval reproduction), or relative pitch (5% were poor-pitch interval singers without tending to transpose pitch). Altogether, these findings suggest that there may be a variety of phenotypes of poor-pitch singing in the general population, which is likely to reflect malfunctioning of different components of the song system (Pfordresher and Brown, 2007; Dalla Bella et al., 2011; Hutchins and Peretz, 2012).

The identification and characterization of phenotypes of poor-pitch singing is particularly relevant for uncovering the functional components of the song system and their relations. In addition, a better understanding of the functional origins of poor-pitch singing is a key element for devising targeted training strategies to limit these difficulties (e.g., during development). Presently, however, we do not dispose of a set of tasks and analysis methods for uncovering and characterizing phenotypes of poor-pitch singing in the general population. Different tasks, spanning from single pitch-matching to imitation and production from memory of complex pitch sequences, are used in various experiments. Moreover, analyses methods and metrics of singing proficiency differ from one study to the other. In some studies, singing accuracy is assessed via peer judgments (e.g., Hébert et al., 2003; Schön et al., 2004; Wise and Sloboda, 2008). In others, singing accuracy is measured objectively using acoustical methods (e.g., Murayama et al., 2004; Terao et al., 2006; Dalla Bella et al., 2007, 2009; Pfordresher et al., 2010). Even when singing proficiency is measured based on acoustical methods, different metrics can be extracted (e.g., pitch interval deviation, signed note error, absolute note error, note accuracy and precision, interval accuracy and precision, contour errors, pitch errors, and so forth; Dalla Bella et al., 2007; Pfordresher and Brown, 2007; Pfordresher et al., 2010).

Unfortunately, to date, there is no consensus about the criterion to adopt for teasing apart poor from good singers (for a review, see Dalla Bella et al., 2011). In some cases participants are considered as poor-pitch singers if their productions deviate from a target pitch (e.g., in a pitch-matching task) by more than a fixed value (i.e., accuracy). This value may vary from one semitone (100 cents = 1/12 of an octave; e.g., Pfordresher and Brown, 2007; Pfordresher et al., 2010), to a quarter of tone (50 cents; Demorest and Clements, 2007; Hutchins and Peretz, 2012). In the first case, the criterion reflects the fact that the semitone is the smallest difference between two neighboring notes in Western music. A problem inherent in the 1-semitone criterion, however, is that it yields a 200-cent acceptable range around the target pitch (e.g., see Hutchins et al., 2012). The 50-cent criterion implies that if a note deviates from a target tone by more than ½ of a semitone it will be likely heard as the other note instead of the target. The choice of this more stringent criterion is thus justified by perceptual factors; indeed, 50 cents is a viable estimate of the acceptability threshold for a tone in a melody to be considered in tune, at least for listeners with musical experience (see Hutchins et al., 2012, for a discussion). Another possibility is to adopt a variable criterion, knowing that accuracy can vary depending on the tested population (e.g., musicians vs. non-musicians) and on the specific task. Poor-pitch singers can thus be identified with respect to a control/comparison group, as is often the case with patients after brain damage (e.g., Schön et al., 2004; Satoh et al., 2007). Alternatively, poor-pitch singers can be those individuals who are outliers in a given group, for example deviating from the average of the group by more than 2 *SD* (e.g., Dalla Bella and Berkowska, 2009). This variable criterion is similar to the one adopted in previous studies to classify individuals suffering from music perception and memory disorders (i.e., congenital amusia) based on the results of the Montreal Battery of Evaluation of Amusia (Peretz et al., 2003). In addition, the definition of impairment based on the deviation of an individual performance from a given population average by 2 *SD* is common in clinical psychology, for example in standardized batteries of tests (e.g., WAIS, WISC, Stanford-Binet Intelligence Scales).

In addition, there is hardly an agreement on the measures of singing proficiency which allow to characterize an individual as a poor-pitch singer. Both absolute pitch differences (i.e., the deviation of produced pitches from the target pitch, in pitch-matching tasks; e.g., Pfordresher and Brown, 2007) and relative pitch differences (i.e., the deviation of produced intervals from the target interval in singing from memory or imitation tasks; e.g., Dalla Bella et al., 2007) can serve to define poor-pitch singing. Moreover, accuracy in producing or imitating pitches is often considered for identifying poor-pitch singers (Dalla Bella et al., 2007; Pfordresher and Brown, 2007; Pfordresher et al., 2010). Accuracy indicates how close the produced pitch or interval is to the target based on the notation. Larger deviation indicates low accuracy. However, treating low pitch accuracy as the main indicator of poor-pitch singing may be misleading. The prevalence of poor-pitch singing is higher when precision is considered instead of accuracy (Pfordresher et al., 2010). Precision refers to the consistency in the repetition of the same pitch class (for absolute pitch) or of the same interval class (for relative pitch). Low consistency is synonymous of low precision. In sum, different criteria and different measures (e.g., accuracy vs. precision) can lead to discordant estimates of the prevalence of poor-pitch singing in the population thus making the comparison of results from different studies an arduous task.

In this study we propose a new tool for assessing singing proficiency (the Sung Performance Battery, SPB), with the goal of providing a comprehensive set of tasks and analysis methods for detecting poor-pitch singers in the general population and characterizing different phenotypes of poor-pitch singing. In doing so, we compared different criteria and various measures of poor-pitch singing. The SPB is formed by 5 tasks, after an assessment of participants' vocal range: (1) single-pitch matching, (2) pitch-interval matching, (3) novel-melody matching, (4) singing from memory of familiar melodies (with lyrics and on a syllable), and (5) imitation of familiar melodies (with lyrics and on a syllable) at a slow tempo indicated by a metronome. The SPB was tested on a group of 50 occasional singers. The renditions were analyzed using acoustical methods. In order to provide the same metric across all the tasks, data was analyzed so as to obtain measures of accuracy and precision in terms of both absolute pitch and relative pitch, as previously suggested by Pfordresher et al. (2010). In addition, poor-pitch singers were identified using both fixed (i.e., 100, and 50 cents) and variable criteria (i.e., 2 *SD* from the average of the group) for comparison.

## Method

### Participants

Fifty occasional singers (35 females and 15 males), aged between 19 and 39 years (*M* = 25.1 years) took part in the Experiment for class credit. Most were university students. None of the participants had received formal musical training. Only three participants received private music lessons during 2–6 years. No participants reported past or present hearing problems or articulatory disorders.

### Materials and procedure

All participants were submitted to a battery of tests for assessing their sung proficiency (SPB). The SPB includes five tasks, preceded by an assessment of vocal range using an adaptive staircase procedure. This assessment is crucial, as it allows to present the stimuli to be imitated (i.e., in pitch-, interval-, and melody-matching tasks) within the vocal range of the participants. To estimate the vocal range, participants are asked first to produce a comfortable pitch on the vowel /a/. Its fundamental frequency is extracted in real time using the YIN algorithm (de Cheveigné and Kawahara, 2002) and used as a point of departure for determining the upper and lower boundaries of the vocal range. To estimate the upper boundary, the participant is asked to imitate isolated notes presented in ascending order starting from the comfortable pitch (step = 3 semitones). When, according to the experimenter, imitation is not possible anymore, lower pitches are presented (i.e., point of reversal) for imitation (step = 2 semitones). When imitation is possible at one of the lower pitches, higher pitches are then presented (reversal) for imitation (step = 1 semitone). For the procedure to stop, three additional reversals (with staircases having a step of 1 semitone) are needed. The upper boundary of the vocal range corresponds to the average pitch height at these last three reversals. The same procedure is used to estimate the lower boundary with the difference that pitches to be imitated were presented at first in a descending order starting from the comfortable pitch. Each note lasts 3 s and is presented twice with a 2-s Inter-Stimulus-Interval (ISI). The participants are asked to listen to the first presentation of each note, to sing along with the second presentation, and finally to produce its pitch. This last production is considered by the experimenter for assessing the vocal range.

#### Task 1 (single-pitch matching)

In this task the participant is asked to imitate 12 target notes of the chromatic scale taken from an octave centered around the midpoint of her/his vocal range. Each note is repeated in two separate trials, for a total of 24 trials. Notes are presented in random order. In a trial, each note (duration = 3 s) is presented twice with an ISI of 2 s. After the second presentation the participant imitates the note as accurately as possible on the vowel /a/. The note is produced twice in a row with a short pause in between.

#### Task 2 (pitch-interval matching)

The participant is presented with 25 target intervals generated from the pitches taken from an octave centered around the midpoint of her/his vocal range. The set of stimuli includes all the intervals spanning from a minor second (e.g., C–C#) through the octave (*n* = 12), presented with an ascending and descending pitch direction (overall, *n* = 24), and the unison. The notes in an interval last 1.6 s each, and the ISI between the two notes is 0.58 s. Intervals are presented in random order. In a trial, each interval is presented twice with an ISI of 2 s. After the second presentation the participant is asked to imitate the interval as accurately as possible on the vowel /a/. The interval is produced twice with a short pause in between.

#### Task 3 (novel-melody matching)

**Figure 1:**
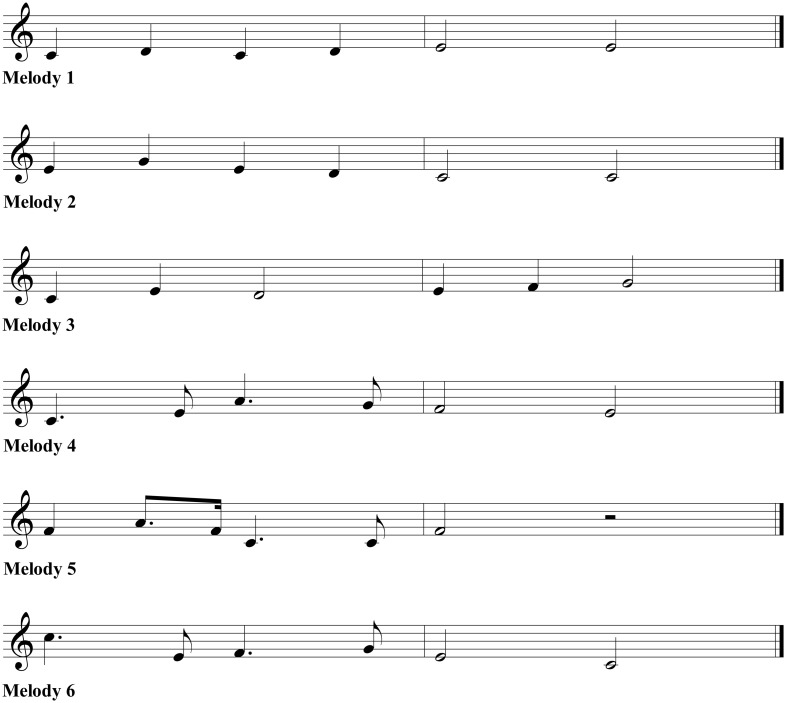
**Score of novel melodies used in Task 3 of the SPB**.

In this task, the participant is asked to imitate six novel six-note melodies, as illustrated in Figure 1. The melodies are centered around the midpoint of the estimated vocal range. Each melody is presented twice with a pause of 4 s. After the second presentation, the participant has to imitate the melody as accurately as possible in terms of both pitch and durations using the syllable /la/.

#### Task 4 (singing from memory of familiar melodies—with lyrics and on a syllable)

**Figure 2:**
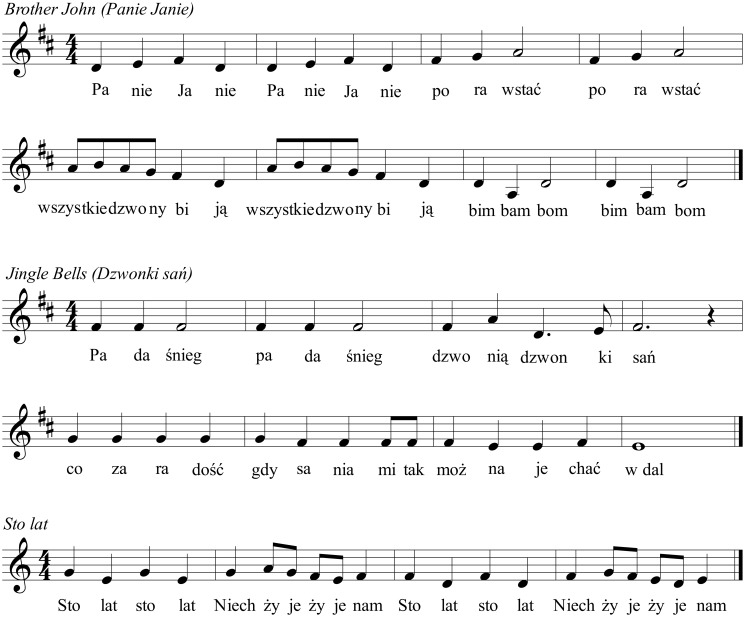
**Score of familiar melodies used in Tasks 4 and 5 of the SPB**.

The participant is asked to sing from memory the beginning of three well-known songs with Polish lyrics (Woźny, 1958; Malko, 1992; Piątek, 2005): the full melody (32 notes) of “Brother John” (*Panie Janie*), the first 8 bars (25 notes) of “Jingle Bells” (*Dzwonki sań*), and the first 4 bars (20 notes) of “Sto lat” (i.e., a familiar Polish melody typically sung at birthdays). The melodies are illustrated in Figure 2. Both starting pitch and tempo are chosen by the participant. Each melody is sung both with lyrics and on the syllable /la/. Written lyrics are made available to the participant during the task.

#### Task 5 (singing of familiar melodies at a slow tempo—with lyrics and on a syllable)

In this task the same song fragments produced in Task 4 are imitated by the participant at a fixed slow tempo. The presentation of each melody is preceded by a metronome indicating the beat (*Brother John*, 96 beats/min, quarter-note IOI = 625 ms; *Jingle Bells*, 125 beats/min, quarter-note IOI = 480 ms; *Sto lat*, 80 beats/min, quarter-note IOI = 750 ms). Four metronome beats are sounded prior to the melody, then the melody is presented twice together with the metronome. Finally, the metronome is turned off and the participant repeats the melody immediately afterwards as accurately as possible. The melodies are presented within the vocal range of individual participants. Moreover, written lyrics are provided during the task.

Before the SPB, a 10-min warm-up session was carried out in which participants sang 3 well-known Polish songs (*Pieski małe dwa*, *Szła dzieweczka*, and *Wlazł kotek*). The SPB and the procedure for computing the vocal range were run on Matlab 7.1. All the pitches presented in the SPB were synthesized complex tones formed by three partials, and characterized by a quadratic up-ramp (duration = 77 ms, for a note lasting 500 ms), and an exponential decay (duration = 433 ms). This sound envelope, used in all the tasks of the SPB, was selected in pilot tests for its voice-like character and conduciveness to vocal imitation. Stimuli were presented over Sennheiser EH2270 headphones at a comfortable level. Vocal performance was recorded with a Shure SM58 microphone on a Fostex D2424LV digital recorder (sampling frequency = 44.1 KHz) and subsequently dumped onto an IBM-compatible computer using Audition Software for further analyses. The SPB lasted ~1 h.

### Analyses

The productions obtained from the SPB were analyzed with acoustical methods, which were successfully adopted in previous studies (e.g., Dalla Bella et al., 2007, 2009; Dalla Bella and Berkowska, 2009; Pfordresher et al., 2010). For the purposes of the present study, the analyses were limited to the pitch dimension (for rhythmic analyses, see Dalla Bella et al., 2007). The first step of the analysis consisted in computing the *pitch height* of the produced notes. In Tasks 1 and 2 this was achieved by extracting the fundamental frequency (F0) from the stable portion of the produced pitch using Praat software (Boersma, 2001) with an accurate autocorrelation method (Boersma, 1993; sampling rate = 100 Hz; Gaussian window = 80 ms). In Tasks 3 through 5, given the higher complexity of the text material associated with music (lyrics or repeated syllables), the procedure was more demanding. As done in previous studies (e.g., Dalla Bella et al., 2007, 2009) the acoustical analyses focussed on the vowel groups (e.g., “o” in “sto”); they are the best targets for acoustical analysis, given that vowels carry the maximum of voicing (Murayama et al., 2004). In all tasks, pitch height was estimated by computing the median of the extracted F0.

Note pitch height was submitted to further analyses to compute measures of accuracy and precision in terms of absolute and relative pitch. These measures of singing proficiency have the advantage that they can be easily and consistently computed across the different tasks of the SPB. Moreover, they can be treated as independent measures of singing proficiency and in previous studies have proven as useful for characterizing singing proficiency in the general population (e.g., Pfordresher et al., 2010; for a review, see Dalla Bella et al., 2011). Accuracy and precision were computed separately for *absolute pitch* (i.e., the pitch height of musical notes) and for *relative pitch* (i.e., the difference between two subsequent pitches, or interval), based on the formulas adopted by Pfordresher et al. (2010), but see below for some discrepancies.

*Accuracy* was computed using Equations (1) and (2) below, for absolute and relative pitch, respectively. *S* indicates the fundamental frequency (F0) of a sung pitch (single note or note in an interval), *T* refers to the F0 of the target pitch, and *i* indexes each of the notes/intervals in a sequence of *N*.

(1) $$\text{Accuracy }\left(\text{absolute pitch}\right)=\left|\frac{\sum_{i}^{N}\left(S_{i}-T_{i}\right)}{N}\right|$$

(2) $$\text{ Accuracy }\left(\text{relative pitch}\right)=\left|\frac{\sum_{i}^{N-1}\left(\left|S_{i+1}-S_{i}\right|-\left|T_{i+1}-T_{i}\right|\right)}{N-1}\right|$$

For absolute pitch (pitch height), accuracy indicates the absolute value of the average difference (in cents) between sung and target pitches, regardless of pitch direction (i.e., as to whether the produced pitch was higher or lower than the target). For relative pitch (intervals), accuracy refers to the absolute value of the average difference between sung pitch intervals and target intervals. This measure of accuracy slightly departs from the original study by Pfordresher et al. (2010). In the study, the signed average difference between sung pitch intervals and target intervals was considered, thus indicating if the intervals were compressed or expanded with respect to the targets.

Moreover, there are additional differences between our computation of accuracy for both absolute and relative pitch and the study by Pfordresher et al. (2010). As far as it can be inferred from their description of the analysis methods, in Pfordresher et al. (2010) study, signed averages of single note or interval differences for each tone sequence (i.e., including 5 tones in their case) were averaged to obtain accuracy for a given individual. Hence, opposite tendencies to sing a single pitch “sharp” or “flat”, or to compress or expand intervals in different sequences canceled out when obtaining a measure of individual accuracy. In our study, the absolute difference between sung pitch notes/intervals and target notes/intervals was computed as indicated in Equations (1) and (2) for each tone sequence (i.e., in Tasks 3, 4, and 5); accuracy values across all sequences were then averaged to obtain an individual's accuracy in relative pitch. This measures of accuracy are preferred here since they do not take pitch direction into account, a measure quantified independently by another variable (see below). Yet, because absolute interval differences for each tone sequence do not cancel out when computing an individual's accuracy, this method is likely to yield higher values of accuracy for relative pitch than previously reported by Pfordresher et al. (2010).

*Precision* was obtained using the same procedure as in Pfordresher et al. (2010). The *SD* of the produced F0/interval for a single pitch/interval class was calculated using Equation (3). *M* is the average produced F0 or interval for a given pitch/interval class, *S* is each of produced pitch/interval. Precision is computed separately for each pitch and interval class. The average across all pitch and interval classes is performed to obtain the measures of precision in terms of absolute and relative pitch, respectively, for a given participant.

(3) $$\text{Precision }(\text{pitch/interval class})=\sqrt{\frac{\sum_{i}^{N(\text{class})}{\left(S_{i}-M_{\text{class}}\right)}^{2}}{N_{\text{class}}}}$$

Even though the main analyses focused on accuracy and precision, additional metrics were computed to compare the results obtained with the SPB with previous studies from our laboratory (e.g., Dalla Bella et al., 2007, 2009). In Tasks 2 through 5 the number of pitch direction/contour errors was computed. Pitch direction was treated as ascending or descending if the sung interval between two notes was higher or lower by more than 1 semitone. A pitch direction/contour error was scored when the pitch direction deviated from the musical score. In Tasks 4 and 5, in addition, pitch interval deviation and the number of pitch interval errors was computed. Pitch interval deviation is a measure of the average size of the pitch deviations, by averaging the absolute difference in semitones between the produced intervals and the intervals prescribed by musical notation. This measure has been treated in previous studies as an alternative measure of interval accuracy (e.g., Dalla Bella et al., 2007, 2009; Berkowska and Dalla Bella, 2009a; Dalla Bella and Berkowska, 2009). However, note that this measure slightly differs from interval accuracy computed by Pfordresher et al. (2010). To obtain pitch interval deviation, before computing the average of the differences between the performed and the target intervals, the absolute value of such differences was calculated; signed differences were maintained, in contrast, to compute interval accuracy. As a result, values of pitch interval deviation are typically larger (i.e., showing worse performance) than interval accuracy. A pitch interval error indicates a produced interval that deviates in magnitude from its respective notated interval. An error was scored when the sung interval was larger or smaller by one semitone than the interval prescribed by the notation. Pitch interval errors were coded irrespectively of pitch direction.

## Results and discussion

### Group performance

From all participants, 3340 complete renditions were recorded across all the tasks (Task1: 1197 performances; Task 2: 1250; Task 3: 297; Task 4: 296; Task 5: 300). Ten performances (0.3% of the entire data set) were discarded due to erroneous or incomplete sound recordings. The participants had a mean vocal range (i.e., difference between the lower and the upper boundaries estimated with the adaptive procedure in the SPB) of ~1 octave and ½ (*M* = 18.52 semitones, *SD* = 4.56 semit.). The extent of the vocal range was not associated with measures of accuracy and precision provided by the SPB. Thus, stimulus presentation in the SPB afforded measures of singing proficiency not biased by singers' vocal range.

Mean and variability for singing accuracy and precision in the five tasks of the SPB, and additional variables (i.e., pitch direction/contour errors, pitch interval deviation, and pitch interval errors) are reported in Tables 1–3. The distribution of pitch accuracy and precision measures for the tested population is often departing from normality and positively skewed (Task 1: mean Shapiro-Wilk *W* = 0.88, *p* < 0.001—Task 2: for relative pitch, mean *W* = 0.89, *p* < 0.001; absolute pitch, accuracy, *W* = 0.71, *p* < 0.001—Task 3: accuracy, mean *W* = 0.76, *p* < 0.001—Task 4: with lyrics, mean *W* = 0.89, *p* < 0.001; with a syllable, accuracy, *W* = 0.73, *p* < 0.001—Task 5: with lyrics, accuracy, relative pitch, *W* = 0.78, *p* < 0.001; absolute pitch, mean *W* = 0.85, *p* < 0.001; with a syllable, relative pitch, mean *W* = 0.84, *p* < 0.001; absolute pitch, mean *W* = 0.82, *p* < 0.001). Hence, the majority of the tested sample was quite accurate and precise, but with some individuals deviating from the majority (individual differences are discussed more in detail below). Precision and accuracy in absolute or relative pitch were typically associated within each task: participants who were very accurate were typically also very precise. This relation was more visible for relative pitch (mean *r* = 0.54, *p* < 0.001) than for absolute pitch (mean *r* = 0.35, *p* < 0.05). In addition, high precision in relative pitch was typically associated to high precision in absolute pitch (in Task 2, *r* = 0.81, *p* < 0.001; Task 3, *r* = 0.65, *p* < 0.001; Task 5, mean *r* = 0.76, *p* < 0.001). This correlation between the results obtained on absolute and relative pitch was less visible when considering accuracy (in Task 2, *r* = 0.45, *p* < 0.001; Task 3, *r* = 0.37, *p* = 0.01; Task 5, mean *r* = 0.11, *p* = ns).

**Table 1:** **Singing proficiency of 50 occasional singers in Tasks 1 and 2 of the SPB**.

**Variable**	**Task 1 (single-pitch matching)**				**Task 2 (pitch-interval matching)**			
	***M* (*SD*)**	**Med**	**Min**	**Max**	***M* (*SD*)**	**Med**	**Min**	**Max**
**ABSOLUTE PITCH**								
Accuracy	140.2 (143.4)	87.8	2.3	522.1	127.1 (163.7)	62.5	6.1	646.5
Precision	64.3 (41.9)	59.5	6.1	183.1	98.1 (50.4)	90.8	10.5	222.1
**RELATIVE PITCH**								
Accuracy	–	–	–	–	99.4 (96.7)	79.7	0.3	398.8
Precision	–	–	–	–	44.8 (28.8)	40.9	4.2	150.0
Direction errors (n)	–	–	–	–	3.1 (3.2)	2.0	0	14.0

*Accuracy and precision are indicated in cents*.

**Table 2:** **Singing proficiency of 50 occasional singers in Task 3 of the SPB**.

**Variable**	**Task 3 (novel-melody matching)**			
	***M* (*SD*)**	**Med**	**Min**	**Max**
**ABSOLUTE PITCH**				
Accuracy	126.4 (145.1)	34.4	16.9	973.3
Precision	78.3 (43.6)	57.0	7.9	217.4
**RELATIVE PITCH**				
Accuracy	40.1 (28.1)	74.7	2.5	103.4
Precision	63.1 (28.8)	72.5	14.9	139.9

*Accuracy and precision are indicated in cents*.

**Table 3:** **Singing proficiency of 50 occasional singers in Tasks 4 and 5 of the SPB**.

**Variable**	**Task 4 (singing familiar melodies from memory)**								**Task 5 (singing familiar melodies—slow tempo)**							
	**Lyrics**				**Syllable**				**Lyrics**				**Syllable**			
	***M* (*SD*)**	**Med**	**Min**	**Max**	***M* (*SD*)**	**Med**	**Min**	**Max**	***M* (*SD*)**	**Med**	**Min**	**Max**	***M* (*SD*)**	**Med**	**Min**	**Max**
**ABSOLUTE PITCH**																
Accuracy	–	–	–	–	–	–	–	–	170.5 (176.3)	126.4	7.8	1006.9	157.9 (177.8)	126.4	5.3	1069.4
Precision	–	–	–	–	–	–	–	–	41.2 (19.9)	39.4	14.9	110.7	36.1 (17.1)	34.7	11.3	70.6
**RELATIVE PITCH**																
Accuracy	26.4 (17.4)	20.7	4.5	77.8	18.9 (14.7)	15.2	1.8	71.3	19.8 (13.5)	14.9	2.5	72.9	17.4 (13.4)	13.4	4.3	80.8
Precision	51.6 (19.6)	49.2	16.0	120.3	41.6 (14.6)	41.3	13.4	80.1	45.4 (15.5)	46.3	20.6	81.7	39.9 (16.1)	39.9	15.1	90.3
Pitch interval deviation	57.3 (25.2)	52.5	18.3	151.5	47.9 (20.9)	45.8	19.1	103.6	49.4 (20.2)	47.2	21.6	113.1	44.2 (19.7)	44.2	6.5	111.5
Contour errors (n)	2.8 (1.8)	2.3	0.7	12.5	2.3 (1.3)	2.0	0.3	6.7	2.4 (1.1)	2.3	0.7	5.7	1.9 (0.9)	1.9	0.7	5.3
Pitch interval errors (n)	4.0 (3.0)	3.3	0	14.0	3.0 (2.3)	2.8	0	9.7	3.1 (2.5)	2.3	0	10.7	2.4 (2.3)	2.4	0	9.7

*Accuracy, precision, and interval deviation are indicated in cents*.

One of the goals of this study was to examine different measures of singing proficiency across a variety of tasks. To this aim, accuracy and precision obtained in each of the five Tasks of the SPB were compared. When accuracy and precision were computed both for absolute and relative pitch (i.e., in Tasks 2, 3, and 5), data were entered in a Pitch (absolute vs. relative) × Measure (accuracy vs. precision) repeated measures ANOVA. The performance of occasional singers was more precise than accurate in Task 1 [*t*_(49)_ = 3.94, *p* < 0.001], and in Task 2 [*F*_(1, 49)_ = 8.77, *p* < 0.01]. In Task 3, the difference between accuracy and precision varied when they were computed based on absolute or relative pitch, as shown by a significant Pitch × Measure interaction [*F*_(1, 49)_ = 12.42, *p* < 0.001]. Occasional singers were more precise than accurate in terms of absolute pitch [*t*_(49)_ = 2.44, *p* < 0.05], but more accurate than precise in terms of relative pitch [*t*_(49)_ = 5.42, *p* < 0.001]. In Task 4, the participants were more accurate than precise in terms of relative pitch, both when singing with lyrics [*t*_(49)_ = 11.78, *p* < 0.001] and on a syllable [*t*_(49)_ = 9.31, *p* < 0.001]. Finally, as observed in Task 3, the difference between accuracy and precision found in Task 5 varied as a function of Pitch [with lyrics, *F*_(1, 49)_ = 41.48, *p* < 0.001; on a syllable, *F*_(1, 49)_ = 33.94, *p* < 0.001]. Greater precision than accuracy was found in terms of absolute pitch [with lyrics, *t*_(49)_ = 5.39, *p* < 0.001; on a syllable, *t*_(49)_ = 4.98, *p* < 0.001], whereas the opposite was observed when considering relative pitch [with lyrics, *t*_(49)_ = 15.40, *p* < 0.001; on a syllable, *t*_(49)_ = 14.64, *p* < 0.001). These findings are partly in keeping with previous results. Pfordresher et al. (2010) showed that occasional singers are more accurate than precise when imitating a short unfamiliar melody and when singing familiar melodies from memory. These tests are akin to Tasks 3 and 4 of the SPB, respectively. With the SPB, when accuracy and precision were calculated based on relative pitch, occasional singers were indeed more accurate than precise (but see interval imitation, Task 2). However, the opposite finding (occasional singers being more precise than accurate) was obtained across all tasks when accuracy and precision were measured based on absolute pitch.

To further probe the effect of the task on measurements of accuracy and precision, repeated measures ANOVAs were run separately for measures of absolute and relative pitch taking Task as the within-subject factor. For absolute pitch, the performances in Tasks 1, 2, 3, and 5 could be compared. Accuracy in terms of absolute pitch did not significantly differ from one task to the other [with lyrics, *F*_(3, 147)_ = 2.45, *p* = ns; with a syllable, *F*_(3, 147)_ = 1.28, *p* = ns]. However, precision was affected by the Task [with lyrics, *F*_(3, 147)_ = 31.28, *p* < 0.001; with a syllable, *F*_(3, 147)_ = 35.59, *p* < 0.001]. Precision was higher in Task 5 as compared to Tasks 1, 2, 3 (Tukey HSD *post-hoc* comparisons, *ps* < 0.001). In addition, precision was lowest when participants imitated intervals (Task 2) as compared to all other tasks (Tukey *ps* < 0.01). For relative pitch, the results obtained in Tasks 2, 3, 4, and 5 were compared. Accuracy in terms of relative pitch varied depending on the Task [with lyrics, *F*_(3, 147)_ = 31.64, ε = 0.38, *p* < 0.001; with a syllable, *F*_(3, 147)_ = 35.17, ε = 0.37, *p* < 0.001]^1^. Accuracy was significantly lower when participants imitated intervals (Task 2), as compared to Tasks 3, 4, and 5 (Tukey *ps* < 0.001). No differences were observed between Tasks 3, 4, and 5. Finally, different degrees of precision in relative pitch were observed in different tasks [with lyrics, *F*_(3, 147)_ = 10.04, ε = 0.78, *p* < 0.001; with a syllable, *F*_(3, 147)_ = 16.89, ε = 0.77, *p* < 0.001]. Precision was the lowest in Task 3 and significantly differed from the values obtained in all the other Tasks (Tukey *ps* < 0.05 − 0.001). Tasks 2, 4, and 5 did not significantly differ with regard to precision in relative pitch. In sum, measures of precision and accuracy vary as a function of the task. Accuracy is more constant than precision across the different tasks, in particular when these measures are computed based on absolute pitch. Precision is particularly sensitive to the task. When this measure is computed based on absolute pitch, differences between the tasks are the most visible (e.g., when comparing imitation of well-known songs at a slow tempo and pitch-interval matching).

Further analyses were performed on the data obtained in Tasks 4 and 5, to examine whether accuracy and precision differ when singing with lyrics vs. singing on a syllable. Singing on a syllable was typically more accurate [relative pitch: Task 4, *t*_(49)_ = 4.15, *p* < 0.001; absolute pitch: Task 5, *t*_(49)_ = 1.98, *p* = 0.05] and more precise [relative pitch: Task 4, *t*_(49)_ = 4.56, *p* < 0.001, Task 5, *t*_(49)_ = 3.17, *p* < 0.01; absolute pitch: Task 5, *t*_(49)_ = 2.45, *p* < 0.05] than singing with lyrics. The advantage of singing on a syllable over singing with lyrics is confirmed when considering the other measures of singing proficiency [pitch interval deviation: Task 4, *t*_(49)_ = 5.00, *p* < 0.001, Task 5, *t*_(49)_ = 3.89, *p* < 0.001; No of contour errors: Task 4, *t*_(49)_ = 2.25, *p* < 0.05, Task 5, *t*_(49)_ = 3.96, *p* < 0.001; No of interval errors: Task 4, *t*_(49)_ = 4.95, *p* < 0.001, Task 5, *t*_(49)_ = 3.18, *p* < 0.01]. These findings confirm previous evidence showing that reducing linguistic information and memory load during singing leads to improved performance in occasional singers (Berkowska and Dalla Bella, 2009a).

### Individual performances (identification of poor-pitch singers)

A further goal of the SPB was to provide a systematic method for uncovering individuals who can be qualified as poor-pitch singers, and for identifying profiles of poor-pitch singers. Given that poor-pitch singing has been defined in the past using different criteria, here three thresholds were considered across the five Tasks of the SPB: two examples of fixed thresholds (100 and 50 cents) and an example of a variable threshold (i.e., performance deviating by more than 2 *SD* from the group average). The number of occasional singers considered as poor-pitch singers according to these criteria, as a function of the task, pitch dimension (absolute vs. relative pitch), and pitch measure (accuracy vs. precision), is presented in Table 4. As can be seen, measures of absolute pitch are more sensitive than measures of relative pitch to poor-pitch singing. In particular, accuracy in imitating target pitches is more efficient than precision in detecting poor-pitch singers in Task 5. In general, the 50-cent criterion is the most sensitive whereas the 2 *SD* criterion is the least sensitive for classifying poor-pitch singers based on absolute pitch. When considering occasional singers' performance in terms of relative pitch, again the 50-cent criterion appear as being the most sensitive to uncover poor-pitch singing. Yet, the 100-cent criterion is definitely insufficient for detecting inaccurate and imprecise singing (e.g., in particular in Tasks 4 and 5). The 2 *SD* criterion is more sensitive than the 100-cent criterion, thus allowing to detect a greater number of poor-pitch singers based on relative pitch. As can be seen, poor-pitch singing is more visible when occasional singers imitated single pitches, intervals, or short unfamiliar melodies (Tasks 1, 2, 3) than when they sang familiar melodies (Tasks 4 and 5). The obtained percent of poor-pitch singers in single pitch-matching tasks (Task 1) is in keeping with previous results using the 50-cent criterion (58% as compared to 62% of poor-pitch singers detected based on accuracy in terms of absolute pitch), and the 100-cent criterion (48% as compared to 40%) (Hutchins and Peretz, 2012). Yet, our results deviate from what observed in previous studies using imitation of short melodies and singing of familiar melodies, and a fixed 100-cent criterion. For example, we found more poor-pitch singers based on accuracy in terms of absolute pitch (i.e., 44%) in Task 3 than previously reported by Pfordresher et al. (2010) (i.e., 13%). In contrast, in comparison with the same study, fewer poor-pitch singers were found based on precision in terms of absolute pitch (30 vs. 56%). This discrepancy increased when poor-pitch singers were defined based on the performance in terms of relative pitch. In Task 3 we found 2 and 14% of poor-pitch singers on accuracy and precision, respectively, as compared to 49 and 60% obtained by Pfordresher et al. (2010). A similar difference was observed in Task 4. These discrepancies may be partly related to sample differences but also to the analysis methods and to the procedure adopted in the SPB. For example, the incidence of poor-pitch singing based on accuracy may have been boosted in some cases because we computed each individual's accuracy irrespective of pitch direction (see Method for details). In some cases, in contrast, given the differences among occasional singers in terms of vocal range, adapting stimulus presentation in imitation tasks to the vocal range of individual participants may have facilitated the task.

**Table 4:** **Number of poor-pitch singers defined according to three different criteria (accuracy or precision >50 cents, >100 cents, >2 SD from the group mean) based on the results of the SPB**.

**Variable**		**Task 1**	**Task 2**	**Task 3**	**Task 4**		**Task 5**	
	**Threshold**				**Lyrics**	**Syllable**	**Lyrics**	**Syllable**
	**(Cents)**	***n* (%)**	***n* (%)**	***n* (%)**	***n* (%)**	***n* (%)**	***n* (%)**	***n* (%)**
**ABSOLUTE PITCH**								
Accuracy	50	29 (58)	29 (58)	39 (78)	–	–	36 (72)	37 (74)
	100	24 (48)	15 (30)	22 (44)	–	–	29 (58)	27 (54)
	2*SD*	3 (6)	4 (8)	1 (2)	–	–	1 (2)	1 (2)
Precision	50	31 (62)	42 (84)	36 (72)	–	–	13 (26)	10 (20)
	100	7 (14)	22 (44)	15 (30)	–	–	1 (2)	0 (0)
	2*SD*	2 (4)	3 (6)	1 (2)	–	–	2(4)	2 (4)
**RELATIVE PITCH**								
Accuracy	50	^−^	28 (56)	17 (34)	5 (10)	4 (8)	2 (4)	2 (4)
	100	–	20 (40)	1 (2)	0 (0)	0 (0)	0 (0)	0 (0)
	2*SD*	–	3 (6)	1 (2)	4 (8)	4 (8)	2(4)	2 (4)
Precision	50	–	15 (30)	32 (64)	24 (48)	12 (24)	19 (38)	9 (18)
	100	–	3 (6)	7 (14)	1 (2)	0 (0)	0 (0)	0 (0)
	2*SD*	–	3 (6)	1 (2)	2 (4)	2 (4)	2 (4)	2 (4)

To illustrate more in detail the different profiles of poor-pitch singing, individual performances on all the tasks of the SPB were reported in Table 5. To this aim, the variable criterion (i.e., accuracy or precision in each Task >2 *SD* from the group mean) was preferred to a fixed criterion. The variable criterion has the advantage that it adapts to the distribution of singing abilities of the tested population, which can vary depending on musical training and on culture. This approach is common in clinical psychology, whereas a disorder is defined relative to a normative group. In addition, a variable criterion appears as more appropriate than a fixed criterion to account for poor-pitch singing across tasks and measures (e.g., precision vs. accuracy). For example, the incidence of inaccurate and imprecise singers vary in the general population. Whereas a 100-cent criterion may be appropriate and justifiable for detecting poor-pitch singing based on accuracy, it may be too stringent when considering precision [see Pfordresher et al. (2010)]. In addition, average accuracy and precision vary as a function of the task, as observed above. A variable criterion allows to adjust to differences in the distribution of singing abilities in the general population as a function of the tasks and of measurements. Finally, another advantage of the variable criterion is that it provides a rather low number of individuals showing impaired singing. This contrasts with the incidence of poor-pitch singing based on other fixed criteria, which in some cases (e.g., with the 50-cent criterion) is unrealistically high (more than 70% when poor-pitch singing is determined based on absolute pitch).

**Table 5:** **Profiles of poor-pitch singing based on the SPB, ranked from the most inaccurate/imprecise to the least inaccurate/imprecise separately for absolute and relative pitch**.

**Participant**	**Task 1**		**Task 2**		**Task 3**		**Task 4**				**Task 5**			
							**Lyrics**		**Syllable**		**Lyrics**		**Syllable**	
	**Accuracy**	**Precision**	**Accuracy**	**Precision**	**Accuracy**	**Precision**	**Accuracy**	**Precision**	**Accuracy**	**Precision**	**Accuracy**	**Precision**	**Accuracy**	**Precision**
**ABSOLUTE PITCH**														
o30	**522.1**	89.3	**646.5**	**204.2**	272.9	110.7	**–**	**–**	**–**	**–**	476.2	43.0	482.7	**70.6**
o69	**491.2**	112.3	404.9	106.3	**973.3**	112.7	**–**	**–**	**–**	**–**	**1006.9**	32.8	**1069.4**	17.7
o33	**443.9**	43.9	**511.2**	120.5	180.5	108.6	**–**	**–**	**–**	**–**	459.1	61.1	448.5	**70.3**
o27	243.4	**181.6**	94.5	170.1	112.5	**217.4**	**–**	**–**	**–**	**–**	261.0	72.3	215.9	40.2
o42	123.6	**183.1**	8.6	174.9	84.4	110.6	**–**	**–**	**–**	**–**	231.7	**110.7**	194.8	58.6
o47	392.5	43.6	**614.9**	**204.8**	290.9	100.7	**–**	**–**	**–**	**–**	407.7	68.1	361.3	69.3
o32	367.6	61.3	**488.4**	104.8	101.9	36.8	**–**	**–**	**–**	**–**	296.9	39.3	217.1	39.2
o59	9.2	67.1	195.7	87.7	127.9	55.5	**–**	**–**	**–**	**–**	279.3	**92.2**	220.0	68.0
o64	354.4	53.8	224.4	**222.1**	103.0	159.9	**–**	**–**	**–**	**–**	53.3	38.2	63.9	64.2
**RELATIVE PITCH**														
o42	**–**	**–**	116.0	**114.8**	65.3	55.3	**70.6**	75.5	**54.9**	**71.5**	**72.9**	**81.7**	**80.8**	**90.3**
o27	**–**	**–**	126.4	**107.6**	67.3	87.0	**77.8**	83.9	**64.5**	56.7	**66.7**	69.2	33.2	50.1
o32	**–**	**–**	**348.9**	43.6	39.7	101.2	**65.9**	**120.3**	15.2	42.5	17.2	53.5	15.6	38.5
o62	**–**	**–**	**398.8**	32.4	94.6	47.2	**74.9**	73.9	**71.3**	46.9	28.7	69.1	39.4	71.6
o64	**–**	**–**	211.2	**149.9**	**103.4**	**139.9**	21.7	42.3	22.0	42.8	38.1	57.1	41.1	69.8
o59	**–**	**–**	**300.1**	61.8	87.8	57.0	42.0	72.2	**59.1**	48.3	30.9	59.6	13.7	48.5
o31	**–**	**–**	4.8	14.0	43.1	75.6	54.8	**98.9**	7.5	63.5	17.2	52.1	50.2	**50.2**
o36	**–**	**–**	59.7	34.2	51.1	115.9	39.5	58.9	15.6	40.9	36.3	**81.3**	21.6	40.9
o6	**_–_**	**_–_**	23.1	49.5	4.1	42.1	12.1	29.8	8.5	13.4	9.5	39.0	26.9	**82.7**
o23	**–**	**–**	1.1	39.7	23.8	54.1	12.8	49.7	12.9	**80.1**	10.6	53.6	8.6	39.2

*Poor-pitch singers were defined using a variable criterion (accuracy or precision in each Task > 2 SD from the group mean). Values of accuracy and precision are reported in bold when they exceeded this criterion. In addition, the codes of participants who were poor-pitch singers in terms of both absolute and relative pitch are underlined*.

With this criterion, 14 poor-pitch singers were identified overall. Five of them (o27, o42, o32, o59, and o64) exhibited inaccurate singing in terms of both absolute and relative pitch. Four (o30, o33, o47, o69) were selectively inaccurate when they had to reproduce the appropriate pitch height but were still accurate in reproducing the target intervals across all tasks. Five (o6, o23, o31, o36, o62) revealed the opposite profile. In spite of generally accurate and precise reproduction of target pitches, these poor singers had difficulties in imitating and producing the target intervals. Most poor-pitch singers showed impairments both in terms of accuracy and precision across the different tasks (o27, o30, o32, o33, o42, o47, o59, o64). However, the productions of 2 poor-pitch singers (o62, o69) was selectively inaccurate without being imprecise. Four participants presented the opposite pattern, by showing accurate but imprecise performances. In sum, the analysis of accuracy and precision obtained from the different tasks of the SPB allows to uncover different patterns of poor-pitch singing. This finding is consistent with previous suggestions that the breakdown of the song system, due to malfunctioning of specific elements of the network underlying proficient singing, can bring about a variety of phenotypes of poor-pitch singing (e.g., Pfordresher and Brown, 2007; Dalla Bella et al., 2011; Hutchins and Peretz, 2012).

## Conclusions

The goal of this study was to present and evaluate the SPB, a new tool for systematically assessing singing proficiency in the general population. The SPB, tested in a group of 50 occasional singers, provided measures of accuracy and precision on a set of 5 tasks, based on the ability to reproduce target pitches (i.e., absolute pitch) or target intervals (i.e., relative pitch) presented isolately, or in the context of novel and familiar melodies. Occasional singers are more accurate and precise when imitating or reproducing from memory well-known songs than when imitating target pitches, intervals, or short novel melodies, in keeping with previous evidence (Pfordresher et al., 2010). In addition, occasional singers are systematically more accurate and more precise when singing well-known melodies on a syllable than with lyrics. This finding may result from reduced linguistic memory load when singing on a syllable. In this condition, singers can focus on the retrieval of melodic information, thus leading to improved pitch matching and enhanced production of pitch intervals. Rhythmic factors may also play a role. Indeed, in previous studies this advantage when singing on a syllable was associated to reduced temporal variability, probably due to the regularization effect of repeating the same linguistic unit (Berkowska and Dalla Bella, 2009a; see also Dalla Bella et al., 2012). Whether improved accuracy and precision when singing on a syllable depend on enhanced rhythmic performance deserves further enquiry.

Two fixed (50, 100-cent) criteria and one variable criterion (2 *SD* from the group average) used in previous studies to identify poor-pitch singers were applied to the results obtained with the SPB. The results confirmed the prevalence of poor-pitch singing previously obtained with single pitch-matching (Hutchins and Peretz, 2012). Nevertheless, we found discrepancies relative to Pfordresher et al. (2010) when considering the performance for novel and familiar melodies. Occasional singers are not systematically less precise than accurate, as previously indicated by Pfordresher and collaborators. This is true when considering interval production in a melodic context, but not when imitating isolated intervals. Moreover, in terms of absolute pitch, it was found that occasional singers are more precise than accurate. The causes of these discrepancies may lie in the details of the analyses methods and in the testing procedures (e.g., stimulus presentation within the vocal range) adopted in the SPB.

After comparing the results from the three cut-offs, the variable criterion has been preferred over the fixed criteria (see above for the reasons of this choice). With this criterion, 14 poor-pitch singers were identified (i.e., 28% of the tested sample). Moreover, the SPB was successful in uncovering important individual differences among poor-pitch singers. Double dissociations have emerged when comparing singing proficiency in terms of absolute or relative pitch, and accuracy vs. precision. Deficient imitation of the target pitch height is not necessarily accompanied by impaired reproduction or target intervals, and vice versa. Moreover, inaccurate singing does not always go hand in hand with imprecise singing. This finding sheds light on different phenotypes of poor-pitch singing, thereby suggesting that this disorder is not monolithic. The ability to sing fractionates as a result of a developmental anomaly. This suggests that the mechanisms underlying absolute/relative pitch production, and underpinning accuracy and precision may enjoy some degree of functional independence. The locus of these distinct processes (e.g., whether they affect sensorimotor translation or perceptual/motor planning processes) remains to be specified in the vocal sensorimotor loop. In addition, describing different profiles of poor-pitch singing is highly relevant as they contribute to enrich the discussion around the causes of poor-pitch singing. Several studies converge in indicating that there are multiple possible causes of poor-pitch singing, including deficient perception, poor motor control, timbral-translation errors, deficient sensorimotor mapping, and memory disorders (Pfordresher and Brown, 2007; Berkowska and Dalla Bella, 2009b; Dalla Bella et al., 2011; Hutchins and Peretz, 2012). Thus, it is not totally surprising that this diversity reflects in a variety of poor-pitch singing profiles. The phenotypes uncovered with the SPB suggest that there may be distinct sources of impairment depending on the mechanisms regulating pitch accuracy and precision, and for the computation of pitch height/intervals.

The SPB has a few advantages which make it an ideal instrument for uncovering cases of poor-pitch singing. It includes a few core tasks which are representative of the paradigms adopted across most studies and which tap basic abilities underlying vocal performance, such as imitation of simple sequences, complex novel and well-known melodies, and singing from memory. In addition, the battery provides an assessment of singing proficiency grounded in objective acoustical analysis, used systematically across all the tasks of the SPB. For example, the results obtained with the SPB indicated that pitch accuracy and precision vary depending on the task, and on whether absolute or relative pitch dimensions are taken into account. This fact has important consequences for uncovering cases of poor-pitch singing, especially when the classification of inaccurate or imprecise singing is based on just one task, or on a single measure. Someone can perform accurately and precisely on one task (e.g., singing well-known melodies from memory), but still reveal poor-pitch singing in another task (e.g., matching pitch intervals) or when a different measure of singing proficiency is considered. A reliable classification of poor-pitch singing rather requires a multidimensional and systematic assessment of the various dimensions of pitch production (absolute vs. relative pitch, and accuracy vs. precision). The SPB responds to this need. Given its sensitivity to different patterns of poor-pitch singing the battery is ideally suited for testing functional hypotheses about the structural elements of the song system (e.g., whether the control of singing accuracy and precision is subserved by independent neuronal networks), and for shedding light on the causes of poor-pitch singing. Finally, a systematic and thorough assessment of singing abilities paves the ground to the development of successful aiding strategies for poor-pitch singers (e.g., based on imitation, Tremblay-Champoux et al., 2010). Nonetheless, the SPB presents also a few limits. By focussing exclusively on pitch accuracy and precision the battery does not allow to assess the role of timbre on imitation, in spite of the fact that this aspect can play a role in poor-pitch singing (Hutchins and Peretz, 2012). Moreover, in order to provide a complete assessment of vocal sensorimotor abilities, the SPB could be complemented by perceptual tasks tailored to the vocal tasks (e.g., assessing discrimination of single pitches, intervals, novel and familiar melodies).

### Conflict of interest statement

The authors declare that the research was conducted in the absence of any commercial or financial relationships that could be construed as a potential conflict of interest.

## Footnotes

^1^The Greenhouse-Geisser correction for inhomogeneity of variance was applied whenever appropriate. Uncorrected degrees of freedom, epsilon value, and probability level following correction are reported.
