# Corrigendum: Uncovering phenotypes of poor-pitch singing: the Sung Performance Battery (SPB)

**DOI:** 10.3389/fpsyg.2016.01460

**Published:** 2016-09-30

**Authors:** Magdalena Berkowska, Simone Dalla Bella

**Affiliations:** ^1^Department of Cognitive Psychology, University of Finance and Management (WSFiZ) in WarsawWarsaw, Poland; ^2^Movement to Health Laboratory (EuroMov), University of Montpellier 1Montpellier, France; ^3^Institut Universitaire de FranceParis, France

**Keywords:** music cognition, music disorders, singing, tone-deafness, amusia

In the original article, we neglected to thank our sponsor Narodowe Centrum Nauki, grant number Dec-2011/01/N/HS6/03986 to Magdalena Berkowska. The authors apologize for this oversight. This error does not change the scientific conclusions of the article in any way.

## Author contributions

All authors listed, have made substantial, direct, and intellectual contribution to the work, and approved it for publication.

### Conflict of interest statement

The authors declare that the research was conducted in the absence of any commercial or financial relationships that could be construed as a potential conflict of interest.

